# Supplementary material for: Hearing sensitivity and amplitude coding in bats are differentially shaped by echolocation calls and social calls
Source: Proc Biol Sci. 2021 Jan 6;288(1942):20202600. doi: 10.1098/rspb.2020.2600 (PMC7892409; doi:10.1098/rspb.2020.2600)
Supplement: Additional methods, results, and raw data [file rspb20202600supp1.pdf]

Electronic Supplementary Material for

Hearing sensitivity and amplitude coding in bats are differentially shaped by echolocation calls and social calls

Lattenkamp, Ella Z.*; Nagy, Martina; Drexl, Markus; Vernes, Sonja C.; Wiegrebe, Lutz; Knörnschild, Mirjam*

*corresponding authors: mirjam.knoernschild@mfn.berlin; ella.lattenkamp@evobio.eu

Content

	Pages
**Additional methods**	
ABR setup	2
Figure S1: ABR setup	3
Anaesthetics application	4
Table S1: Sample sizes, injected anaesthesia combination drug and anaesthesia duration for ABR measurements	4
**Additional results**	
Figure S2: High frequency resolution ABR contour plots for 18 individual bats from 11 species	5
Figure S3: Species-specific hearing thresholds of 11 species	6
Figure S4: Echolocation calls and isolation calls of bats from 4 families	7
Figure S5: Sex-specific ABR thresholds for 4 species	8
Figure S6: Phylogenetic trees, regression slopes, and maximum likelihood parameter estimates of the phylogenetic comparative analyses	9
Table S2: Acoustic parameters of echolocation calls and social vocalizations from 11 focal bat species	10-11
Table S3: Hearing sensitivity peaks and peak frequencies of echolocation and isolation calls for phylogenetic comparative analyses (38 species)	12-14
Stochastic Linear Ornstein-Uhlenbeck models for Comparative Hypotheses (SLOUCH)	15
Table S4: Results of the phylogenetic comparative analyses	15
Comparison of new ABR data with existing audiograms in the literature	16
**References**	17-22

ABR setup

The ABRs were measured in a small sound-attenuating box (PELI 1450 case, Peli products, CA, USA; inner dimensions: 37.1 x 25.8 x 15.2 cm^3, **Fig. S1a**). The box was first lined with a fine copper mesh to reduce electrical interferences, and then lined with sound-attenuation foam, reducing reverberations within the box (**Fig. S1a**). The bats were positioned frontally facing the loudspeaker (R2004/602000, ScanSpeak, Videbæk, Denmark), which was embedded in the side of the box (**Fig. S1b**). The measured bat species varied in size and ear shape. Thus, the bats were always placed in a way that their outer ear opening was placed at a 4 cm distance from the loudspeaker. The varying sizes of the bats were compensated for with different heights of moveable soft foam pads, so that the heads of the bats were always placed on one level with the horizontal centre of the loudspeaker. The speaker was connected to an amplifier (M032N, Kemo® Electronic, Germany), which was powered via a 4-battery pack (NCR18650GA batteries, Li-Ion, 3.6V, 3500 mAh, Panasonic/Sanyo, Ottobrunn, Germany). Both stimulus presentation and ABR recording were done by an audio interface (ADI-2 PRO FS, RME, Haimhausen, Germany), running at a sampling rate of 384 kHz.

The sound system of the setup was calibrated with a 1/8" measuring microphone (B&K4138 without protective grid, Bruel & Kjaer, Bremen, Germany) connected to a measuring amplifier (B&K Measuring Amplifier Type 2636, Bruel & Kjaer, Bremen, Germany). The acoustic impulse response of the loudspeaker was measured at the bats' head position within the setup using broadband (2-140 kHz) noise. From the measured acoustic impulse response, a compensation impulse response was generated. The used stimuli were convolved with the compensational impulse response in order to present them with a linear phase and a flat frequency response between 3 and 120 kHz.

Figure S1

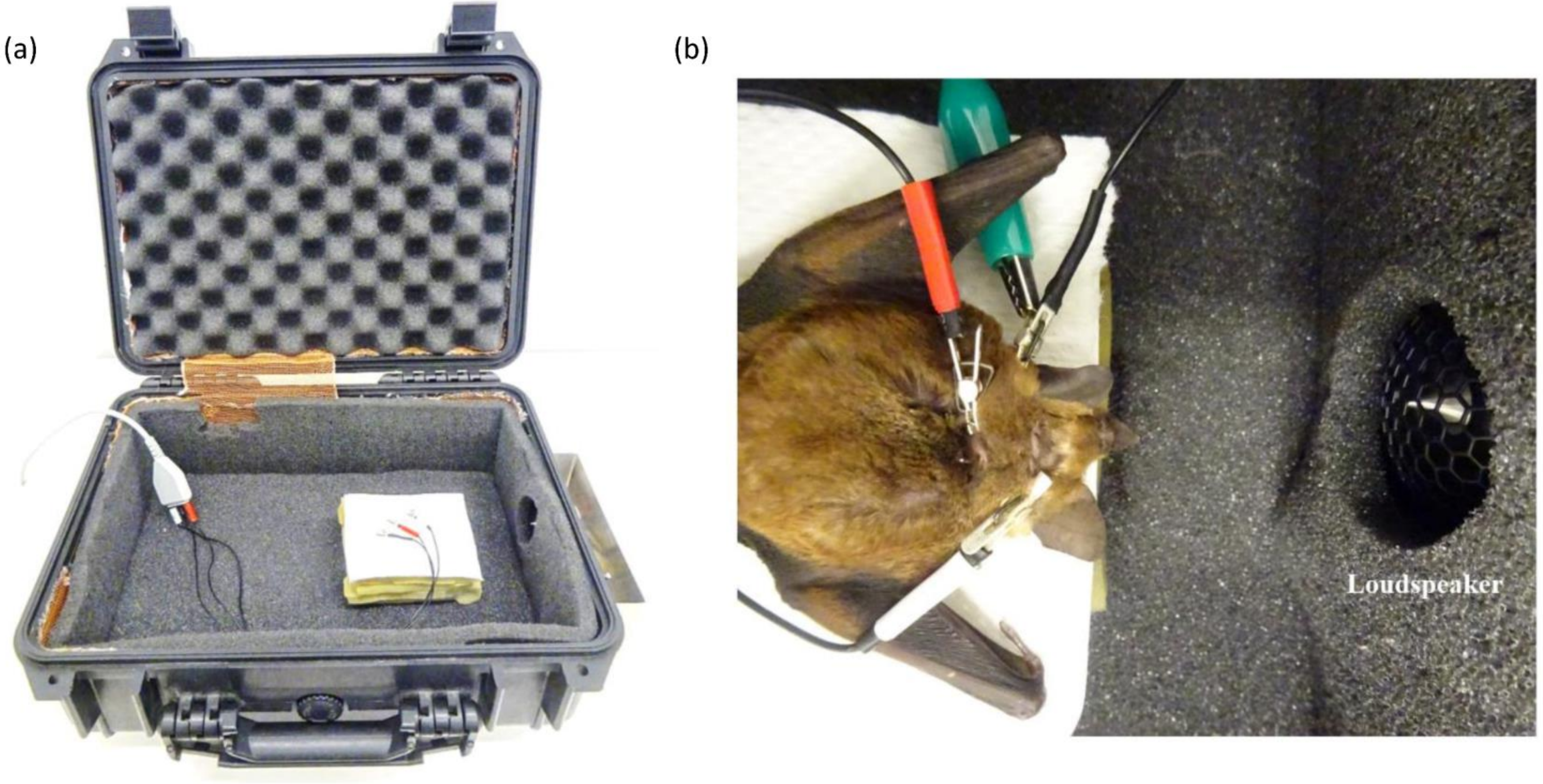

Figure S1: ABR setup. **(a)** Picture of the mobile ABR setup in the open configuration. The loudspeaker is embedded in the right side of the box. The setup is lined first with a copper mesh to reduce electrical disturbance (see gap between lid and bottom part of the box) and then covered in sound absorbing foam. The electrode cable is lead into the box via a cut-out gap in the left side of the box. The foam pads are positioned in front of the loudspeaker and covered with a paper towel. **(b)** Positioning of the bats in the setup. The bats were positioned frontally facing the loudspeaker with a distance of 4 cm between the loudspeaker and their outer ear openings. Different heights of moveable soft foam pads were used to compensate for the different sizes of the measured species. The heads of the bats were always placed on one level with the centre of the loudspeaker. The recording electrode (red) was placed at the caudal midline of the head, close to the brainstem. The reference electrode (white) was placed at the dorsal midline of the head between the ears. The ground electrode (black) was placed on the base of the left ear of the animal (or the wing or tail membrane). The ground electrode was connected to the copper lining of the setup via a crocodile clamp (green) in order to ground the animal.

Anaesthetics application

Bats were anesthetized with a combination drug of Medetomidine, Midazolam, and Fentanyl at least 10 minutes before the experiment (average administered amounts per species are listed in **Table S1**). The depth of anaesthesia was quantified by triggering the inter-toe reflex by mechanical stimulation (slight pinching with fine forceps). The drug was injected subcutaneously between the shoulder blades (needle: 0.45 x 12 mm). The reactions of the different bat species to the drug varied strongly, thus in some cases a second dose needed to be injected after 10-15 minutes. The anaesthesia was not antagonized after the measurements were concluded and thus varied in its duration strongly between the different species (**Table S1**). Subsequent to the ABR measurements, some individuals (N=28) were sacrificed for a different experiment (not reported in this study; MiAmbiente permit SE/A-3-19, STRI ACUC protocol 2019-0302-2022). For these individuals, natural anaesthesia durations are not known. During anaesthesia, eye cream (Bepanthen®, 5% Dexpanthenol, Bayer AG, Leverkusen, Germany) was applied to prevent the eyes from drying out. Bats were kept in a room with ambient temperature for the whole procedure. After waking up from anaesthesia, we offered each individual food and water and released it at the site of capture.

Table S1: Numbers of adult bats measured per species, weight, amount of injected fentanyl per gram body weight, and duration of the anaesthesia [time from injection until first reactions to sounds could be noted (i.e. ear movements)].

Family	Species	Males	Females	Total # bats	Weight [g]	Injected fentanyl [µg per g bodyweight]	Anaesthesia duration [min] (N)
Emballonuridae	*Saccopteryx bilineata*	7	5	12	7.2 ± 0.6	0.032 ± 0.007	144 ± 31 (9)
	Saccopteryx leptura	1	3	4	5.2 ± 0.7	0.030 ± 0.008	69 (1)
	Rhynchonycteris naso	4	1	5	3.4 ± 0.4	0.029 ± 0.016	
Molossidae	*Molossus molossus*	1	5	6	9.8 ± 0.6	0.031 ± 0.006	336 ± 77
Mormoopidae	*Pteronotus parnellii*	8	1	9	22.7 ± 1.0	0.009 ± 0.001	280 ± 126
Phyllostomidae	*Carollia perspicillata*	13	0	13	18.6 ± 1.3	0.011 ± 0.005	124 ± 90 (12)
	Desmodus rotundus	3	3	6	36.2 ± 1.4	0.017 ± 0.005	
	Glossophaga soricina	3	3	6	10.1 ± 0.7	0.009 ± 0.001	297 ± 34 (3)
	Phyllostomus hastatus	7	0	7	118.2 ± 9.2	0.008 ± 0.002	574 ± 269 (6)
Thyropteridae	*Thyroptera tricolor*	3	7	10	4.6 ± 0.7	0.018 ± 0.005	362 ± 238 (5)
Vespertilionidae	*Myotis nigricans*	8	0	8	3.8 ± 0.3	0.061 ± 0.018	372 ± 186 (7)

Measurements are given as means (± standard deviation). Several individuals were used in an additional experiment after the acquisition of the ABRs and their natural anaesthesia durations are not known. The sample size (N) for the calculation of the anaesthesia duration is given in brackets if they deviate from the total sample size for this species. For the anaesthesia combination drug, 0.1 mg fentanyl corresponds to 10 mg Midazolam and 1 mg Medetomidin.

Figure S2

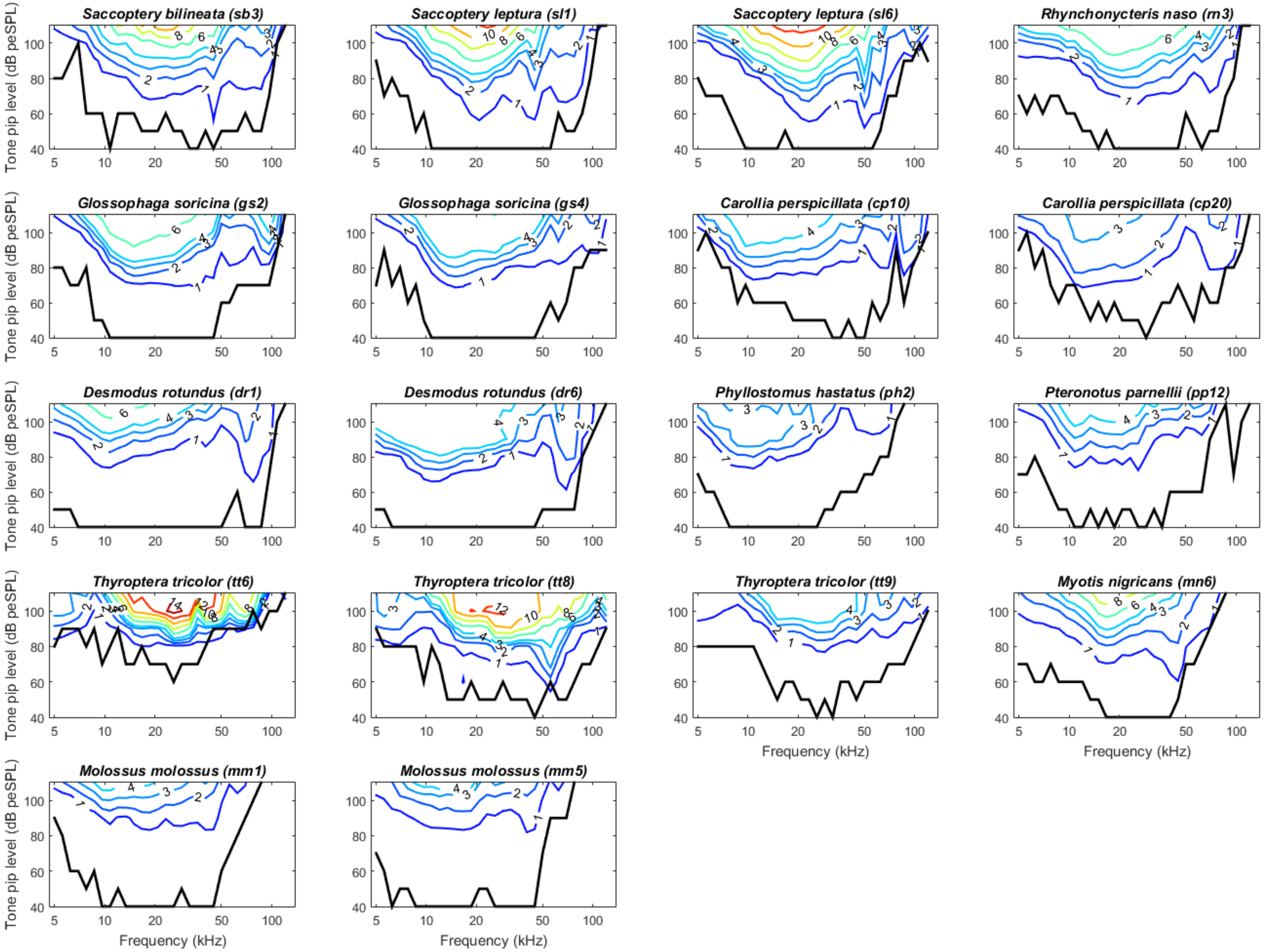

Figure S2: High frequency resolution ABR contour plots for individual bats (N=18). The mean ABR threshold per species is depicted (black line). The isoresponse lines represent the strength of the ABR signal (colours and numbers indicate µV response strength). These additional ABR measurements were conducted with a 30-step log frequency resolution between 5 and 120 kHz. The minimum stimulus presentation level was 40 dB peSPL. Some bats had ABRs with thresholds below this minimum level, which is represented by the horizontal level cut-off at 40 dB.

Figure S3

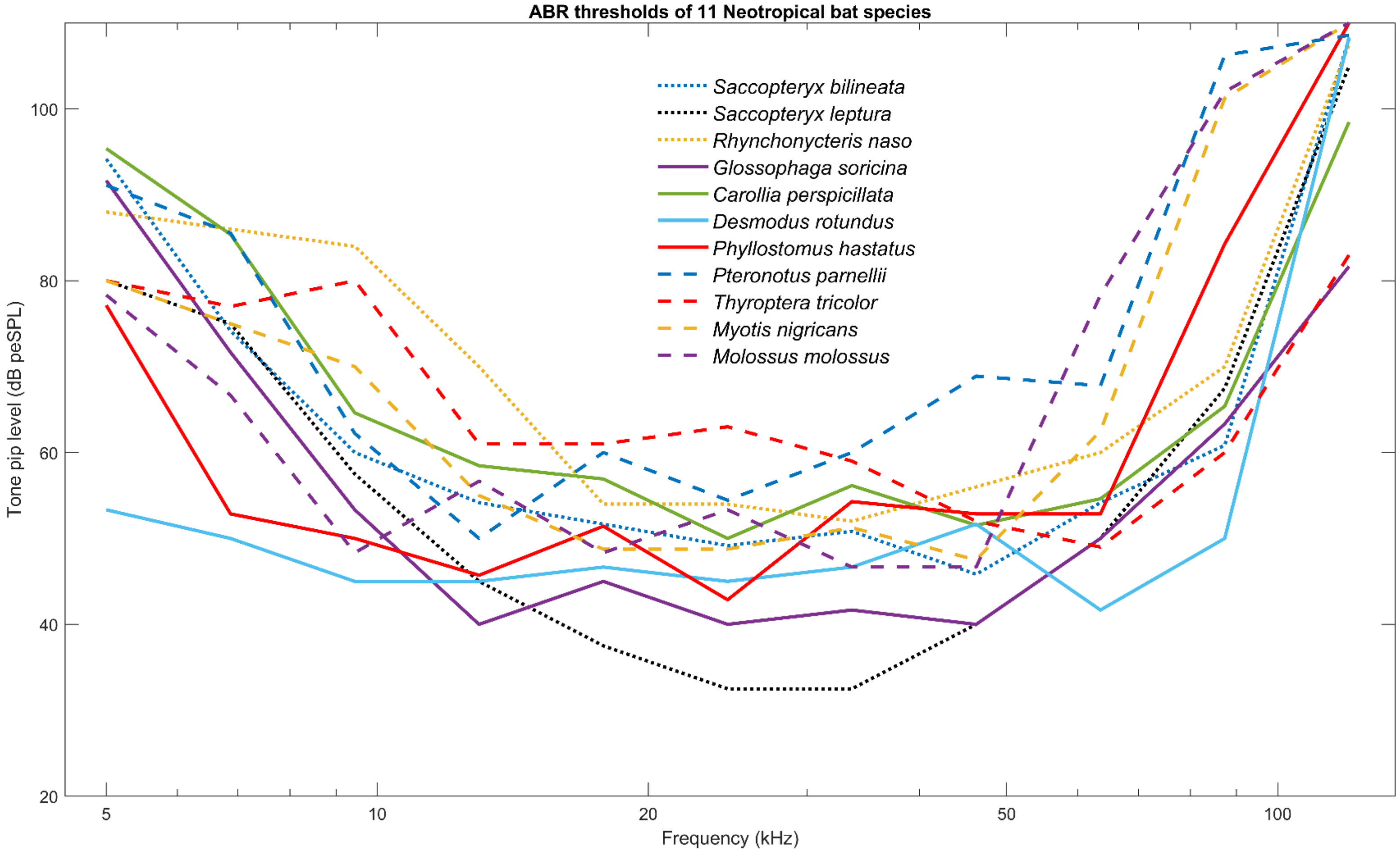

Figure S3: Comparative summary figure illustrating all eleven species-specific mean hearing thresholds. Colours indicate the different species and line styles indicate family (finely dotted lines: Emballonuridae; solid lines: Phyllostomidae; widely dotted lines: Mormoopidae, Thyropteridae, Vespertilionidae, and Molossidae). Despite the species-specific shape of the thresholds, a general trend of sensitivity increase between 5-15 kHz and decrease above 50-60 kHz is noticeable.

Figure S4

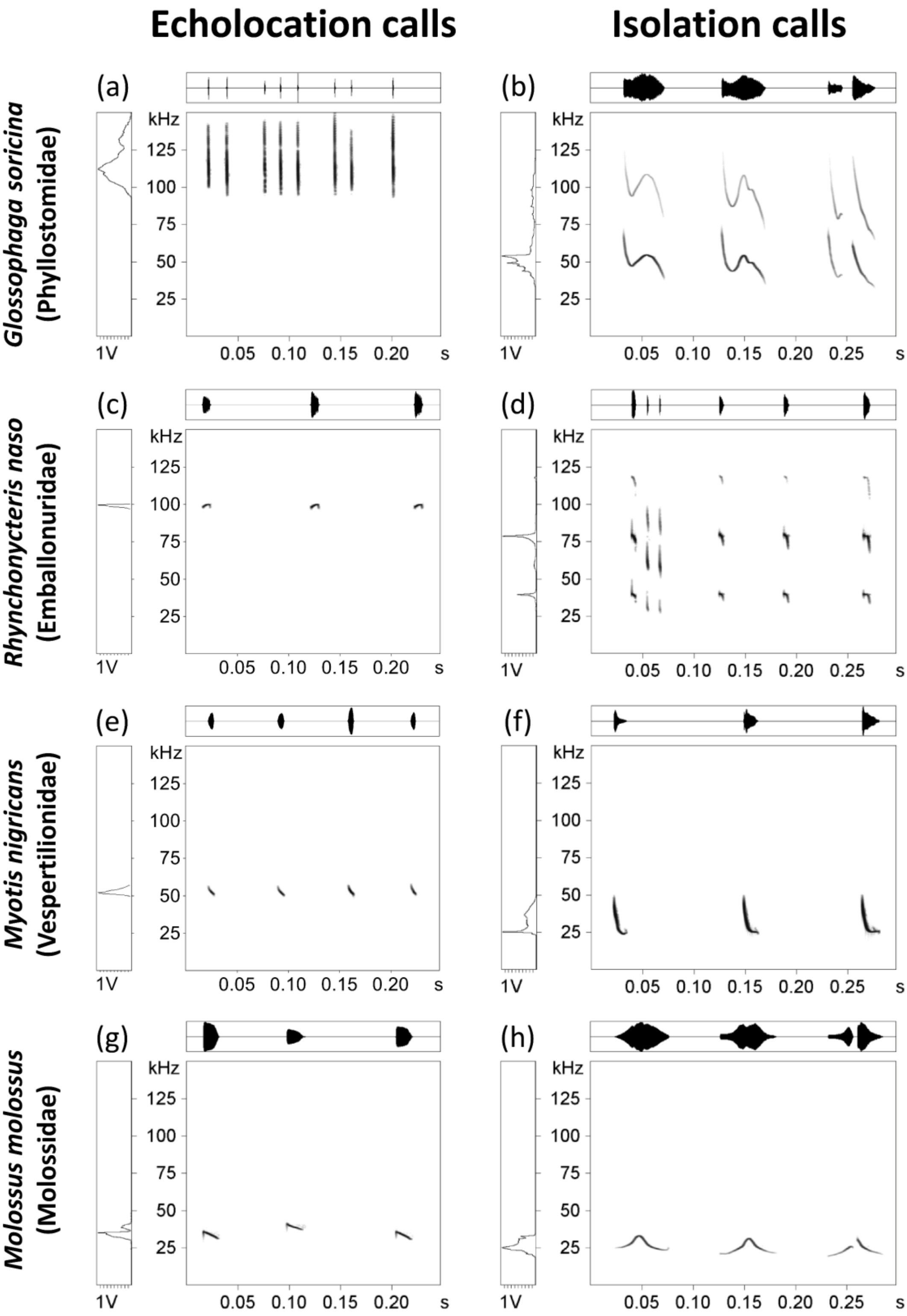

Figure S4: Echolocation calls and isolation calls of bats from four families. Oscillograms (top of the graphs), sonograms (bottom centre) and power spectra (bottom left) illustrate the difference in spectro-temporal structure between both vocalization types. Echolocation calls and isolation calls of *Glossophaga soricina* (**a-b**), *Rhynchonycteris naso* (**c-d**), *Myotis nigricans* (**e-f**) and *Molossus molossus* (**g-h**) are shown. Vocalizations were recorded by MK. Sonograms were created with a 1024-point FFT and a Hamming Window with 87.5% overlap (sampling rate: 300 kHz, resolution: 16 bit).

Figure S5

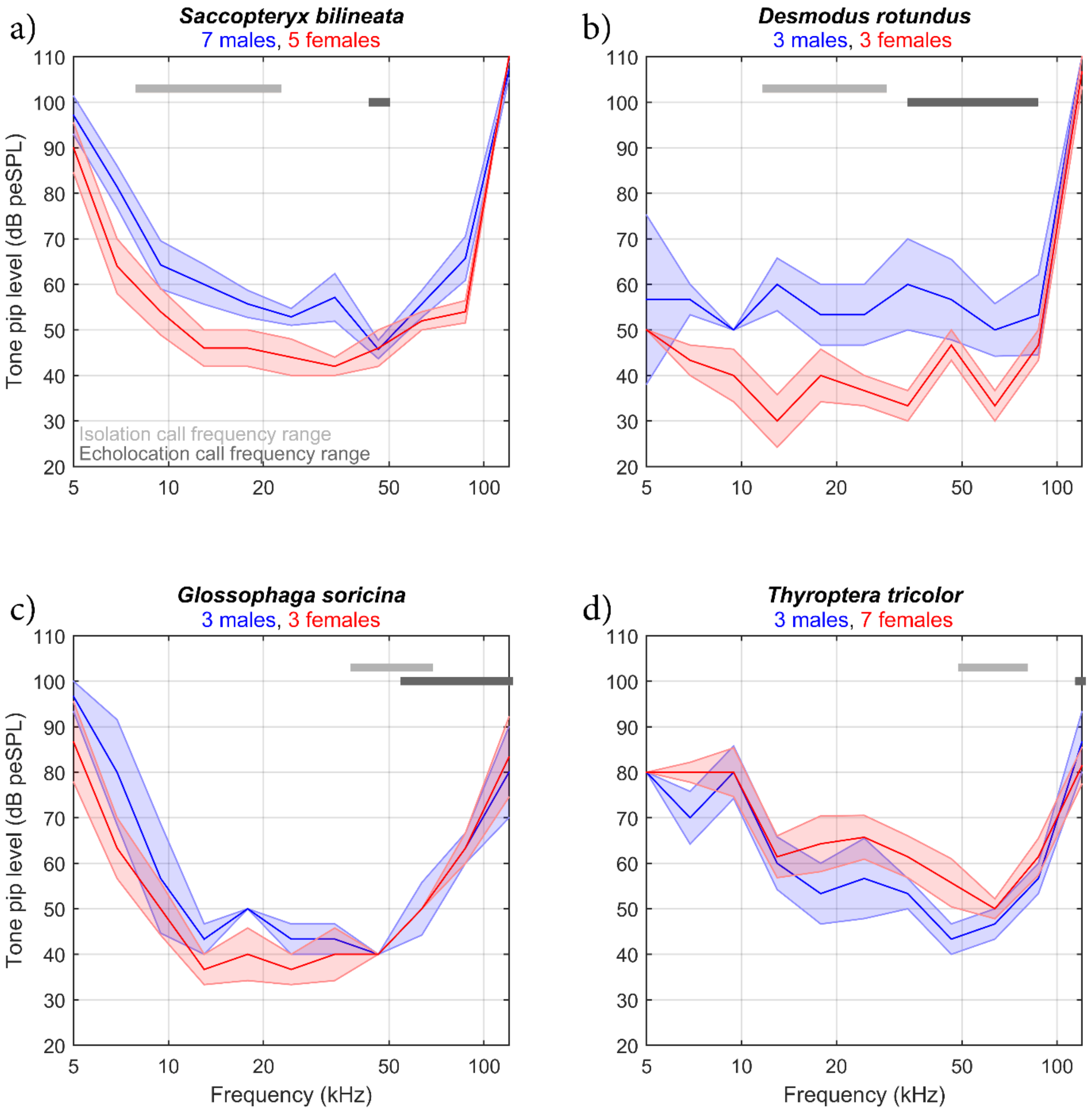

Figure S5: Sex-specific ABR thresholds with 95% CI. For four species, at least three individuals per sex were measured. ABR thresholds for female *Saccopteryx bilineata* (a) and *Desmodus rotundus* (b) were noticeably lower than ABR thresholds for males. Female *Glossophaga soricina* (c) showed the same trend, but the thresholds of both sexes greatly overlapped. *Thyroptera tricolor* (d), on the other hand, showed generally more sensitive thresholds for males than females, although thresholds overlapped considerably as well. Light grey bars indicate the frequency range of species-specific isolation calls, while dark grey bars indicate the frequency range of echolocation calls.

Figure S6

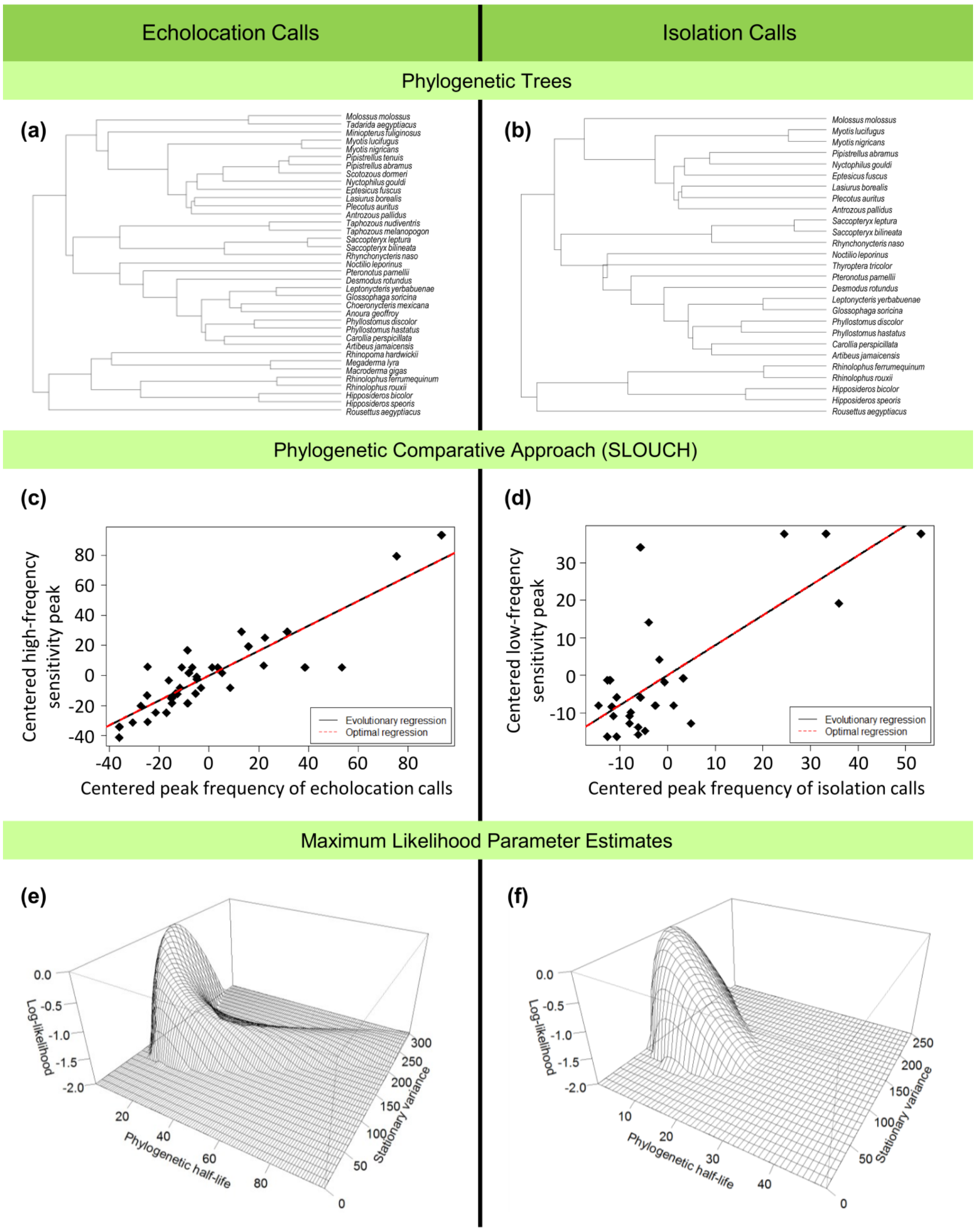

Figure S6: Phylogenetic trees, regression slopes, and maximum likelihood parameter estimates of the phylogenetic comparative analyses for two datasets: **(a, c, e):** Echolocation calls; **(b, d, f):** Isolation calls. **(a, b):** Phylogenetic trees were assembled using a recent molecular phylogeny (Amador et al. 2016). **(c, d):** Results of phylogenetic comparative analyses depict a positive relation between hearing sensitivity peaks (traits) and peak frequency of calls (predictor). Predictors and traits were centred in each regression model. The optimal regression slope describes the expected relationship between hearing sensitivity and calls' peak frequency if no constraints on the evolution towards the optimal state existed (i.e. phylogenetic inertia). The evolutionary regression slope depicts the current relationship between hearing sensitivity and calls' peak frequency. In most scenarios, the slope of the evolutionary regression is shallower than the slope of the optimal regression; the difference between optimal and evolutionary regression is influenced by the speed of adaptation. In our case, however, both regression slopes are similar, suggesting a very fast adaptation and a correspondingly weak phylogenetic signal. **(e, f):** Support surfaces show maximum likelihood parameter estimates for phylogenetic half-lives and stationary variances obtained by phylogenetic comparative analyses. Phylogenetic half-life is tied to tree length and indicates when half the evolution towards the optimum is reached (the larger half-life becomes, the stronger is the phylogenetic signal). Stationary variance is Brownian motion around the evolutionary optimum. Elevated plot areas show all points within two support units (= log-likelihood) of the best estimates.

Table S2: Acoustic parameters [frequency range (min./max.) and peak frequency (pf)] of echolocation calls and social vocalizations from focal bat species.

Species	min. freq [kHz]	max. freq [kHz]	peak freq [kHz]	Data sources
**Echolocation calls**				
Saccopteryx bilineata	44.3	48.9	45.5-47.2	Knörnschild et al 2012a
Saccopteryx leptura	50.8	56.0	51.3-54.6	Jung et al 2007
Rhynchonycteris naso	95.0	102.0	98.2	pf: Jung et al 2007; min/max: Barquero 2010
Molossus molossus	33.5	42.8	35-41.5	Jung et al 2014
Myotis nigricans	50.9	61.5	54.2	Siemers et al 2001
Thyroptera tricolor	117.6	182.4	147.0	I. Geipel unpubl.
Pteronotus parnellii	53.3	61.2	59.8	G. Gessinger unpubl.
Phyllostomus hastatus	40.0	80.0	46.0	Bohn et al 2004
Desmodus rotundus	34.6	84.6	72.6	Rodríguez-San Pedro & Allendes 2017
Carollia perspicillata	58.0	102.0	91.0	pf: Brinklov et al 2011; min/max: Thies et al 1998
Glossophaga soricina	56.0	137.0	113.1	pf: Gessinger unpubl.; min/max: Simon et al 2006
**Isolation calls**				
Saccopteryx bilineata	8.10	22.10	11.20	Knörnschild et al 2012b
Saccopteryx leptura	10.87	27.46	13.60	M. Knörnschild unpubl.
Rhynchonycteris naso	70.67	88.01	78.94	M. Knörnschild unpubl.
Molossus molossus	19.66	29.42	23.12	M. Knörnschild unpubl.
Myotis nigricans	24.71	43.38	26.95	M. Knörnschild unpubl.
Thyroptera tricolor	50.09	78.43	58.98	G. Chaverri unpubl.
Pteronotus parnellii	8.00	14.00	10.00	pf: Kanwal unpubl. [cited in Clement & Kanwal 2012]; min/max: Vater et al 2003
Phyllostomus hastatus	10.00	20.00	15.00	pf: Bohn et al 2004; min/max: Bohn et al 2007
Desmodus rotundus	12.00	28.00	13.00	pf: Bohn et al 2006; min/max: Schmidt & Manske 1973
Carollia perspicillata	22.90	43.40	30.60	Knörnschild et al 2013
Glossophaga soricina	38.80	66.80	50.20	Engler et al 2017

Table S2 (continued): Acoustic parameters [frequency range (min./max.) and peak frequency (pf)] of echolocation calls and social vocalizations from focal bat species.

Species	min. freq [kHz]	max. freq [kHz]	peak freq [kHz]	Data sources
**Courtship calls / songs**				
Saccopteryx bilineata	17.73	47.14	30.20	M. Knörnschild unpubl.
Saccopteryx leptura	12.53	28.73	22.97	M. Knörnschild unpubl.
Rhynchonycteris naso	29.70	99.10	73.70	M. Knörnschild unpubl.
Molossus molossus	11.93	41.13	29.63	M. Knörnschild unpubl.
Carollia perspicillata	11.05	36.64	13.75	Knörnschild et al 2014
Glossophaga soricina	44.82	82.87	58.83	M. Knörnschild unpubl.
**Contact calls**				
Thyroptera tricolor	15.69	35.60	24.44	Gillam & Chaverri 2012; Chaverri & Gillam 2010 (inquiry call)
Thyroptera tricolor	49.15	65.34	56.83	Gillam & Chaverri 2012; Chaverri & Gillam 2010 (response call)
Phyllostomus hastatus	4.70	11.50	6.70	Boughman 1997 (screech call)
Desmodus rotundus	19.30	36.20	25.40	Carter et al 2012
**Territorial song and alert calls**				
Saccopteryx bilineata	11.00	19.91	14.92	Knörnschild et al 2010 (territorial song)
Glossophaga soricina	24.28	68.51	25.58	M. Knörnschild unpubl. (alert call type 1)
Glossophaga soricina	11.98	65.87	17.40	M. Knörnschild unpubl. (alert call type 2)

Only focal species for which we generated ABR data ourselves are listed here. Acoustic parameters were extracted from own data and the literature.

We selected echolocation calls recorded during search flight for parameter extraction. Whenever possible, recordings from the wild were used to minimize the risk of altered call design due to a confined environment in the laboratory. Echolocation calls in our dataset were either constant frequency with or without an optional frequency modulated part (CF or CF-FM), had a quasi-constant frequency part (QCF) or consisted of downward sweeps with varying degrees of frequency modulation (FM). For CF and QCF calls, we reported minimum, maximum, and peak frequencies of the (quasi-) constant frequency parts of the most prominent harmonic. For FM calls, we reported minimum, maximum, and peak frequencies of the whole call, including all present harmonics.

Social call parameters were measured for the whole range of calls (bandwidth). For multisyllabic calls, acoustic parameters were assessed on the level of call, not syllable. We only used isolation calls from non-volant pups and made certain that we did not include echolocation call precursors for parameter extraction. Isolation calls were recorded from hand-held pups in most cases.

Table S3: Hearing sensitivity peaks and peak frequencies of echolocation and isolation calls for phylogenetic comparative analyses.

**Family**	**Species**	**Data for phylogenetic comparative analyses**				**Threshold detection**		**Data sources (own data and literature)**	
		**Sensitivity peak high [kHz]**	**Echolocation call peak freq [kHz]**	**Sensitivity peak low [kHz]**	**Isolation call peak freq [kHz]**	**Type**	**Bat condition**	**Sensitivity peaks**	**Peak frequencies**
Molossidae	*Molossus molossus*	33.7	38.2	17.8	23.1	neuronal (SD)	anesthetized	this study	Jung et al 2014; Knörnschild unpubl.
	Tadarida aegyptiaca	17	23.6	7.5	unknown	neuronal (IC)	awake	Neuweiler et al 1984	Collen 2012
Miniopteridae	*Miniopterus fuliginosus*	46	47	no peak	unknown	neuronal (IC)	anesthetized	Furuyama et al 2018	Hase et al 2016
Vespertilionidae	*Myotis lucifugus*	60	51.6	40	21.7	behavioral	awake	Dalland 1965	Ratcliffe & Dawson 2003; Thomson et al 1985
	Myotis nigricans	46.3	54.2	17.8	27	neuronal (SD)	anesthetized	this study	Siemers et al 2001; Knörnschild unpubl.
	Pipistrellus abramus	43	44,6	20	20	neuronal (IC)	awake	Boku et al 2015	Collen 2012; Hiryu & Riquimaroux 2011
	Pipistrellus tenuis	50	48	20	unknown	neuronal (IC)	awake	Neuweiler et al 1984	Collen 2012
	Scotozous dormeri	55	43.6	no peak	unknown	neuronal (IC)	awake	Neuweiler et al 1984	Collen 2012
	Nyctophilus gouldi *	33.5	42.5	11	21	neuronal (IC)	anesthetized	Guppy & Coles 1988	Collen 2012; Guppy & Coles 1988
	Eptesicus fuscus	64	35	20	15	behavioral	awake	Koay et al 1997	Petrites et al 2009; Monroy et al 2011
	Lasiurus borealis	27.5	35	45	61.7	neuronal (SD)	anesthetized	Obrist & Wenstrup 1998	Obrist & Wenstrup 1998; Schmidt-French et al 2006
	Plecotus auritus *	50	56.5	12	19.5	neuronal (IC)	anesthetized	Coles et al 1989	Water & Jones 1995; deFanis & Jones 1995
	Antrozous pallidus *	40	44.8	15	17.7	neuronal (PC)	anesthetized	Brown et al 1978	Collen 2012; Brown 1976

Table S3 (continued): Hearing sensitivity peaks and peak frequencies of echolocation and isolation calls for phylogenetic comparative analyses.

Family	Species	Data for phylogenetic comparative analyses				Threshold detection		Data sources (own data and literature)	
		Sensitivity peak high [kHz]	Echolocation call peak freq [kHz]	Sensitivity peak low [kHz]	Isolation call peak freq [kHz]	Type	Bat condition	Sensitivity peaks	Peak frequencies
Emballonuridae	*Taphozous nudiventris*	24	23.7	no peak	unknown	neuronal (IC)	awake	Neuweiler et al 1984	Collen 2012
	Taphozous melanopogon	27	29.1	no peak	12	neuronal (IC)	awake	Neuweiler et al 1984	Collen 2012; Gould 1979
	Saccopteryx leptura	63.6	53	24.5	13.6	neuronal (SD)	anesthetized	this study	Jung et al 2007; M. Knörnschild unpubl.
	Saccopteryx bilineata	46.3	46.4	17.8	11.2	neuronal (SD)	anesthetized	this study	Knörnschild et al 2012a; Knörnschild et al 2012b
	Rhynchonycteris naso	63.6	98.2	63.6	79	neuronal (SD)	anesthetized	this study	Jung et al 2007; M. Knörnschild unpubl.
Noctilionidae	*Noctilio leporinus*	57.5	54.6	24	25	neuronal (SD)	awake	Wenstrup 1984	Schnitzler et al 1994; Bohn et al 2006
Thyropteridae	*Thyroptera tricolor*	N/A	147	63.6	59	neuronal (SD)	anesthetized	this study	I. Geipel unpubl.; G. Chaverri unpubl.
Mormoopidae	*Pteronotus parnellii* **	63.6	61	24.5	13	neuronal (SD)	anesthetized	this study	Collen 2012
Phyllostomidae	*Desmodus rotundus* *	87.3	72.6	9.4	13	neuronal (SD)	anesthetized	this study	Rodríguez-San Pedro & Allendes 2017; Bohn et al 2006
	Leptonycteris yerbabuenae	60	64.9	30	24	neuronal (C)	anesthetized	Howell 1974	Collen 2012; Gould 1975
	Glossophaga soricina	63.6	113.1	63.6	50.2	neuronal (SD)	anesthetized	this study	G. Gessinger unpubl.; Engler et al 2017
	Choeronycteris mexicana	75	50.96	10	unknown	neuronal (C)	anesthetized	Howell 1974	Collen 2012
	Anoura geoffroyi	65	81.69	45	unknown	neuronal (C)	anesthetized	Howell 1974	Collen 2012

Table S3 (continued): Hearing sensitivity peaks and peak frequencies of echolocation and isolation calls for phylogenetic comparative analyses.

Family	Species	Data for phylogenetic comparative analyses				Threshold detection		Data sources (own data and literature)	
		Sensitivity peak high [kHz]	Echolocation call peak freq [kHz]	Sensitivity peak low [kHz]	Isolation call peak freq [kHz]	Type	Bat condition	Sensitivity peaks	Peak frequencies
Phyllostomidae (continued)	*Phyllostomus discolor*	63.6	63.2	13	17.7	neuronal (SD)	anesthetized	Linnenschmidt & Wiegrebe 2019	Collen 2012; Esser & Schmidt 1989
	Phyllostomus hastatus	63.6	48.7	9.4	15	neuronal (SD)	anesthetized	this study	Collen 2012; Bohn et al 2004
	Carollia perspicillata	87.3	91	13	30.6	neuronal (SD)	anesthetized	this study	Brinkløv et al. 2011; Knörnschild et al 2013
	Artibeus jamaicensis	56	54.7	16	18	behavioral	awake	Heffner et al 2003	Brinkløv et al 2009; Bohn et al 2006
Rhinopomatidae	*Rhinopoma hardwickii*	38	32.3	19	unknown	neuronal (IC)	anesthetized	Simmons et al 1984	Collen 2012
Megadermatidae	*Megaderma lyra* *	50	68.3	16	unknown	neuronal (IC)	awake	Neuweiler 1989; Rübsamen et al 1988	Collen 2012
	Macroderma gigas *	40	51.1	12	unknown	neuronal (IC)	anesthetized	Neuweiler 1989, Guppy & Coles 1988	Collen 2012
Rhinolophidae	*Rhinolophus ferrumequinum* **	83.5	82.2	17.5	14	physiological	awake	Long & Schnitzler 1975	Collen 2012; Matsumura 1979
	Rhinolophus rouxii **	77.5	75.5	15	14.3	neuronal (IC)	awake	Rübsamen 1987	Rübsamen 1987
Hipposideridae	*Hipposideros bicolor* **	151.5	153	60	20	neuronal (IC)	awake	Neuweiler et al 1984	Collen 2012; Gould 1979
	Hipposideros speoris **	137.5	135	25	29	neuronal (IC)	awake	Neuweiler et al 1984	Schuller 1980; Collen 2012; Habersetzer & Marimuthu 1986
Pteropodidae	*Rousettus aegyptiacus*	45	34.8	10	19.5	behavioral	awake	Koay et al 1998	Holland et al 2004

Data sources for peak frequencies refer first to echolocation calls, second to isolation calls. Electrophysiological detection types for thresholds: subdermal (SD), inferior colliculus (IC), posterior colliculus (PC), cochlear potential (C). * = bats relying on prey-generated sound, ** = CF bats.

Stochastic Linear Ornstein-Uhlenbeck models for Comparative Hypotheses (SLOUCH)

Ornstein-Uhlenbeck models assume that a trait, i.e. hearing sensitivity, evolves towards an optimum that is a linear function of a predictor, i.e. peak frequency of calls. SLOUCH returns an 'optimal regression' slope which describes the expected relationship between the trait and the predictor if no constraints on the evolution towards the optimal state existed (i.e. phylogenetic inertia); it also returns an 'evolutionary regression' slope which depicts the current relationship between the trait and the predictor. In most scenarios, the slope of the evolutionary regression is shallower than the slope of the optimal regression; the difference between optimal and evolutionary regression is influenced by the speed of adaptation. In our case, however, both regression slopes are similar, suggesting a very fast adaptation and a correspondingly weak phylogenetic signal. Stationary variance corresponds to Brownian motion around the evolutionary optimum.

Estimates of phylogenetic half-live and stationary variance should be interpreted with the help of the maximum-likelihood space (see **Figure S5, e-f**).

For the intercept-only model, the estimates of phylogenetic half-live and stationary variance vary considerably when recalculated. However, the estimates always have high values.

Table S4: Results of the phylogenetic comparative analyses (SLOUCH).

Trait	Predictor	N	Phylogenetic half-life	Stationary variance	R^2 [%]	Optimal Regression Slope ± SE (95%-CI)	Evolutionary Regression Slope ± SE (95%-CI)	AICc	logL
Hearing sensitivity at higher frequencies	/	37	2 679,320	18 843,424	/	/	/	329	-161.28
	Peak frequency of adult echolocation calls	37	0.014	165.5951	0.767	0.824 ± 0.074 (0.969-0.679)	0.824 ± 0.074 (1.602 - 0.658)	303	-147.04
Hearing sensitivity at lower frequencies	/	28	7.336	305.673	/	/	/	238	-115.50
	Peak frequency of pup isolation calls	28	1.00E-08	128.880	0.577	0.799 ± 0.131 (1.056-0.542)	0.799 ± 0.131 (1.056-0.542)	218	-103.91

For both the null and the full model, we report the number of species in the analyses (N), the maximum-likelihood estimates of the phylogenetic half-life [in units of tree length] and the stationary variance, the phylogenetically corrected R^2, the estimated slopes of the optimal and evolutionary regression with standard errors (SE) and 95%-CI, the model fit described by the AICc score and the model support based on log likelihood (logL).

Comparison of ABR data with existing audiograms in the literature

We obtained audiograms for 86 bats from 11 Neotropical species by evaluating the ABR signal quantitatively and objectively, i.e. by calculating the signal's rms amplitude and determining its significance via bootstrapping (Linnenschmidt & Wiegrebe 2019). Audiograms for four species measured in the present study were previously published and serve as a reference point for the presented data. ABR thresholds are less sensitive than previously reported behavioural audiograms (between 20-60 dB lower; Kössl 1992; Koay et al. 2002, 2003; Gröger & Wiegrebe 2006; Esser & Eiermann 2008; Heffner et al. 2013). This divergence does not present an issue for our analyses as we focused on qualitatively assessing the bats' hearing sensitivity and not on the extraction of an absolute auditory threshold.

The audiogram of *Desmodus rotundus* was previously measured in two behavioural studies, each testing three individuals (Gröger & Wiegrebe 2006; Heffner et al. 2013). The comparatively low hearing threshold detected for low frequencies (< 10 kHz), which is thought to enable detection of large prey items in these sanguivorous bats (Gröger & Wiegrebe 2006), was shown in both studies and is mirrored in our ABR data. The sensitivity peak of *D. rotundus'* high frequency hearing varies across the different studies, but was in all cases in the range of their echolocation calls (34.6-84.6 kHz; Rodríguez-San Pedro & Allendes 2017).

For *Phyllostomus hastatus,* one behavioural audiogram (Koay et al. 2002) and one neuronal audiogram (recorded from the inferior colliculus; Esser & Eiermann 2008) are available. The high frequency peak of all measurements falls within the echolocation call frequency range (40 – 80 kHz; Bohn et al. 2004). The peak of our ABR threshold and of the first isoresponse line fall between the peaks reported in the behavioural and neuronal audiograms.

For *Carollia perspicillata*, one behavioural audiogram (Koay et al. 2003) and two neuronal audiograms (recorded from the inferior colliculus, IC (Sterbing et al. 1994) and the auditory brainstem (Wetekam et al. 2020)) are available. While our mean ABR threshold peak of *C. perspicillata* is about 20 kHz lower than reported in the behavioural and IC audiograms (at 50 kHz instead of 70 kHz), the first isoresponse line of our ABR data perfectly fits the bandwidth of the species-specific echolocation calls (58- 102 kHz; Brinkløv et al. 2011). Moreover, our ABR data corresponds well to a recently published audiogram of *C. perspicillata* that was obtained with the same method as our audiogram (Wetekam et al. 2020).

For *Pteronotus parnellii*, audiograms obtained with cochlear microphonics, i.e. evoked otoacoustic emissions, are available (Kössl & Vater 1985a, b; Kössl 1994). *Pteronotus parnellii* is the only bat species measured in this study that produces constant frequency (CF) echolocation calls. CF calls have a very small bandwidth, but the echolocation frequencies are individually different and can vary by 35 Hz (Keating et al. 1994). The audiogram of *P. parnellii* shows a sensitivity peak corresponding to the CF call bandwidth, which is very narrow and corresponds to their acoustic fovea (Kössl & Vater 1985a, b; Kössl 1994). In this study, we used a predefined parameter space (see methods), which we tested in all bat species. Although we detected a peak in the ABR threshold, which corresponds to the main CF component of *P. parnellii*, we most likely did not measure at the exact position of the individual CF bandwidth and thus missed the most sensitive point of hearing. Furthermore, the current tone-pips may be spectrally too broad to probe the narrow-band sensitivity changes in *P. parnellii* hearing around their main CF component.

References

Amador L.I., Arévalo R.L.M., Almeida F.C., Catalano S.A., Giannini N.P. 2018 Bat systematics in the light of unconstrained analyses of a comprehensive molecular supermatrix. *J Mamm Evol* **25**(1), 37-70. (doi:10.1007/s10914-016-9363-8).

Barquero Villalobos K. 2010 Patrones de vocalización durante interacciones sociales de *Rhynchonycteris naso* (Chiroptera: Emballonuridae) en Costa Rica [Master Thesis], Universidad de Costa Rica.

Bohn K., Boughman J., Wilkinson G., Moss C. 2004 Auditory sensitivity and frequency selectivity in greater spear-nosed bats suggest specializations for acoustic communication. *J Comp Physiol, A* **190**(3), 185-192. (doi:10.1007/s00359-003-0485-0).

Bohn K.M., Moss C.F., Wilkinson G.S. 2006 Correlated evolution between hearing sensitivity and social calls in bats. *Biol Lett* **2**(4), 561-564. (doi:10.1098/rsbl.2006.0501).

Bohn K.M., Wilkinson G.S., Moss C.F. 2007 Discrimination of Infant Isolation Calls by Female Greater Spear-Nosed Bats, *Phyllostomus hastatus*. *Anim Behav* **73**(3), 423-432. (doi:10.1016/j.anbehav.2006.09.003).

Boku S., Riquimaroux H., Simmons A.M., Simmons J.A. 2015 Auditory brainstem response of the Japanese house bat (*Pipistrellus abramus*). *J Acoust Soc Am* **137**(3), 1063-1068. (doi:10.1121/1.4908212).

Boughman J.W. 1997 Greater spear-nosed bats give group-distinctive calls. *Behav Ecol Sociobiol* **40**(1), 61-70. (doi:10.1007/s002650050316).

Brinkløv S., Jakobsen L., Ratcliffe J.M., Kalko E.K., Surlykke A. 2011 Echolocation call intensity and directionality in flying short-tailed fruit bats, *Carollia perspicillata* (Phyllostomidae). *J Acoust Soc Am* **129**(1), 427-435. (doi:10.1121/1.3519396).

Brinkløv S., Kalko E.K., Surlykke A. 2009 Intense echolocation calls from twowhispering'bats, *Artibeus jamaicensis* and *Macrophyllum macrophyllum* (Phyllostomidae). *J Exp Biol* **212**(1), 11-20. (doi:10.1242/jeb.023226).

Brown P. 1976 Vocal communication in the pallid bat, *Antrozous pallidus*. *Z Tierpsychol* **41**(1), 34-54. (doi:10.1111/j.1439-0310.1976.tb00469.x).

Brown P.E., Grinnell A.D., Harrison J.B. 1978 The development of hearing in the pallid bat, *Antrozous pallidus*. *J Comp Physiol, A* **126**(2), 169-182. (doi:10.1007/BF00666371).

Burkard R., Moss C.F. 1994 The brain-stem auditory-evoked response in the big brown bat (*Eptesicus fuscus*) to clicks and frequency-modulated sweeps. *J Acoust Soc Am* **96**(2), 801-810. (doi:10.1121/1.410318).

Carter G.G., Logsdon R., Arnold B.D., Menchaca A., Medellin R.A. 2012 Adult vampire bats produce contact calls when isolated: acoustic variation by species, population, colony, and individual. *PLoS ONE* **7**(6), e38791. (doi:10.1371/journal.pone.0038791).

Chaverri G., Gillam E.H., Vonhof M.J. 2010 Social calls used by a leaf-roosting bat to signal location. *Biol Lett* **6**(4), 441-444. (doi:10.1098/rsbl.2009.0964).

Clement M.J., Kanwal J.S. 2012 Simple syllabic calls accompany discrete behavior patterns in captive *Pteronotus parnellii*: an illustration of the motivation-structure hypothesis. *Sci World J* **2012**, 128695. (doi:10.1100/2012/128695).

Coles R.B., Guppy A., Anderson M.E., Schlegel P. 1989 Frequency sensitivity and directional hearing in the gleaning bat, *Plecotus auritus* (Linnaeus 1758). *J Comp Physiol, A* **165**(2), 269-280. (doi:10.1007/BF00619201).

Collen A. 2012 The evolution of echolocation in bats: a comparative approach, UCL (University College London).

Dalland J.I. 1965 Hearing sensitivity in bats. *Science* **150**(3700), 1185-1186. (doi:10.1126/science.150.3700.1185).

De Fanis E., Jones G. 1995 Post-natal growth, mother-infant interactions and development of vocalizations in the vespertilionid bat *Plecotus auritus*. *J Zool* **235**(1), 85-97. (doi:10.1111/j.1469-7998.1995.tb05130.x).

Engler S., Rose A., Knornschild M. 2017 Isolation call ontogeny in bat pups (*Glossophaga soricina*). *Behaviour* **154**(3), 267-286. (doi:10.1163/1568539x-00003421).

Esser K.-H., Daucher A. 1996 Hearing in the FM-bat *Phyllostomus discolor*: a behavioral audiogram. *J Comp Physiol, A* **178**(6), 779-785. (doi:10.1007/BF00225826).

Esser K.H., Eiermann A. 1999 Tonotopic organization and parcellation of auditory cortex in the FM-bat *Carollia perspicillata*. *Eur J Neurosci* **11**(10), 3669-3682. (doi:10.1046/j.1460-9568.1999.00789.x).

Esser K.H., Schmidt U. 1989 Mother-infant communication in the lesser spear-nosed bat *Phyllostomus discolor* (Chiroptera, Phyllostomidae)—evidence for acoustic learning. *Ethology* **82**(2), 156-168. (doi:10.1111/j.1439-0310.1989.tb00496.x).

Furuyama T., Hase K., Hiryu S., Kobayasi K.I. 2018 Hearing sensitivity evaluated by the auditory brainstem response in *Miniopterus fuliginosus*. *J Acoust Soc Am* **144**(5), EL436-EL440. (doi:10.1121/1.5079904).

Gillam E.H., Chaverri G. 2012 Strong individual signatures and weaker group signatures in contact calls of Spix's disc-winged bat, Thyroptera tricolor. *Anim Behav* **83**(1), 269-276. (doi:10.1016/j.anbehav.2011.11.002).

Gould E. 1975 Neonatal vocalizations in bats of eight genera. *J Mammal* **56**(1), 15-29. (doi:10.2307/1379603).

Gould E. 1979 Neonatal vocalizations of ten species of Malaysian bats (Megachiroptera and Microchiroptera). *Am Zool* **19**(2), 481-491. (doi:10.1093/icb/19.2.481).

Gröger U., Wiegrebe L. 2006 Classification of human breathing sounds by the common vampire bat, *Desmodus rotundus*. *BMC Biol* **4**(1), 18. (doi:10.1186/1741-7007-4-18).

Guppy A., Coles R.B. 1988 Acoustical and neural aspects of hearing in the Australian gleaning bats, *Macroderma gigas* and *Nyctophilus gouldi*. *J Comp Physiol, A* **162**(5), 653-668. (doi:10.1007/BF01342641).

Habersetzer J., Marimuthu G. 1986 Ontogeny of sounds in the echolocating bat *Hipposideros speoris*. *J Comp Physiol, A* **158**(2), 247-257. (doi:10.1007/BF01338568).

Hase K., Miyamoto T., Kobayasi K.I., Hiryu S. 2016 Rapid frequency control of sonar sounds by the FM bat, *Miniopterus fuliginosus*, in response to spectral overlap. *Behav Processes* **128**, 126-133. (doi:10.1016/j.beproc.2016.04.017).

Heffner R.S., Koay G., Heffner H.E. 2003 Hearing in American leaf-nosed bats. III: *Artibeus jamaicensis*. *Hearing Res* **184**(1-2), 113-122. (doi:10.1016/s0378-5955(03)00233-8).

Heffner R.S., Koay G., Heffner H.E. 2013 Hearing in American leaf-nosed bats. IV: The Common vampire bat, *Desmodus rotundus*. *Hearing Res* **296**, 42-50. (doi:10.1016/j.heares.2012.09.011).

Henson O., Schuller G., Vater M. 1985 A comparative study of the physiological properties of the inner ear in Doppler shift compensating bats (*Rhinolophus rouxi* and *Pteronotus parnellii*). *J Comp Physiol, A* **157**(5), 587-597. (doi:10.1007/BF01351353).

Hiryu S., Riquimaroux H. 2011 Developmental changes in ultrasonic vocalizations by infant Japanese echolocating bats, *Pipistrellus abramus*. *J Acoust Soc Am* **130**(4), EL147-EL153. (doi:10.1121/1.3632044).

Holland R.A., Waters D.A., Rayner J.M. 2004 Echolocation signal structure in the Megachiropteran bat *Rousettus aegyptiacus* Geoffroy 1810. *J Exp Biol* **207**(25), 4361-4369. (doi:10.1242/jeb.01288).

Howell D. 1974 Acoustic behavior and feeding in glossophagine bats. *J Mammal* **55**(2), 293-308. (doi:0.2307/1378999).

Jones G., Siemers B.M. 2011 The communicative potential of bat echolocation pulses. *J Comp Physiol, A* **197**(5), 447-457. (doi:10.1007/s00359-010-0565-x).

Jung K., Kalko E., von, Von Helversen O. 2007 Echolocation calls in Central American emballonurid bats: signal design and call frequency alternation. *J Zool* **272**(2), 125-137. (doi:10.1111/j.1469-7998.2006.00250.x).

Jung K., Molinari J., Kalko E.K. 2014 Driving factors for the evolution of species-specific echolocation call design in new world free-tailed bats (Molossidae). *PLoS ONE* **9**(1), e85279. (doi:10.1371/journal.pone.0085279).

Keating A., Henson O., Henson M., Lancaster W., Xie D. 1994 Doppler-shift compensation by the mustached bat: quantitative data. *J Exp Biol* **188**(1), 115-129.

Knörnschild M., Feifel M., Kalko E.K.V. 2013 Mother-offspring recognition in the bat *Carollia perspicillata*. *Anim Behav* **86**(5), 941-948. (doi:10.1016/j.anbehav.2013.08.011).

Knornschild M., Feifel M., Kalko E.K.V. 2014 Male courtship displays and vocal communication in the polygynous bat *Carollia perspicillata*. *Behaviour* **151**(6), 781-798. (doi:10.1163/1568539x-00003171).

Knörnschild M., Jung K., Nagy M., Metz M., Kalko E. 2012a Bat echolocation calls facilitate social communication. *Proc R Soc Lond B Biol Sci* **279**(1748), 4827-4835. (doi:10.1098/rspb.2012.1995).

Knörnschild M., Nagy M., Metz M., Mayer F., von Helversen O. 2010 Complex vocal imitation during ontogeny in a bat. *Biol Lett* **6**(2), 156-159. (doi:10.1098/rsbl.2009.0685.).

Knörnschild M., Nagy M., Metz M., Mayer F., von Helversen O. 2012b Learned vocal group signatures in the polygynous bat *Saccopteryx bilineata*. *Anim Behav* **84**(4), 761-769. (doi:10.1016/j.anbehav.2012.06.029).

Koay G., Bitter K.S., Heffner H.E., Heffner R.S. 2002 Hearing in American leaf-nosed bats. I: *Phyllostomus hastatus*. *Hearing Res* **171**(1-2), 96-102. (doi:10.1016/s0378-5955(02)00458-6).

Koay G., Heffner H.E., Heffner R.S. 1997 Audiogram of the big brown bat (*Eptesicus fuscus*). *Hearing Res* **105**(1), 202-210. (doi:10.1016/s0378-5955(96)00208-0).

Koay G., Heffner R., Heffner H. 1998 Hearing in a megachiropteran fruit bat (*Rousettus aegyptiacus*). *J Comp Psychol* **112**(4), 371. (doi:10.1037/0735-7036.112.4.371).

Koay G., Heffner R.S., Bitter K.S., Heffner H.E. 2003 Hearing in American leaf-nosed bats. II: *Carollia perspicillata*. *Hearing Res* **178**(1-2), 27-34. (doi:10.1016/s0378-5955(03)00025-x).

Kössl M. 1992 High frequency distortion products from the ears of two bat species, *Megaderma lyra* and *Carollia perspicillata*. *Hearing Res* **60**(2), 156-164. (doi:0.1016/0378-5955(92)90018-I).

Kössl M. 1994 Evidence for a mechanical filter in the cochlea of the 'constant frequency'bats, *Rhinolophus rouxi* and *Pteronotus parnellii*. *Hearing Res* **72**(1-2), 73-80. (doi:10.1016/0378-5955(94)90207-0).

Kössl M., Vater M. 1985a Evoked acoustic emissions and cochlear microphonics in the mustache bat, *Pteronotus parnellii*. *Hearing Res* **19**(2), 157-170. (doi:10.1016/0378-5955(85)90120-0).

Kössl M., Vater M. 1985b The cochlear frequency map of the mustache bat, *Pteronotus parnellii*. *J Comp Physiol, A* **157**(5), 687-697. (doi:10.1007/BF01351362).

Linnenschmidt M., Wiegrebe L. 2019 Ontogeny of auditory brainstem responses in the bat, *Phyllostomus discolor*. *Hearing Res* **373**, 85-95. (doi:10.1016/j.heares.2018.12.010).

Long G.R., Schnitzler H.-U. 1975 Behavioural audiograms from the bat, *Rhinolophus ferrumequinum*. *J Comp Physiol, A* **100**(3), 211-219. (doi:10.1007/BF00614531).

Matsumura S. 1979 Mother-infant communication in a horseshoe bat (*Rhinolophus ferrumequinum nippon)*: development of vocalization. *J Mammal* **60**(1), 76-84. (doi:10.2307/1379760).

Monroy J.A., Carter M.E., Miller K.E., Covey E. 2011 Development of echolocation and communication vocalizations in the big brown bat, *Eptesicus fuscus*. *J Comp Physiol, A* **197**(5), 459-467. (doi:10.1007/s00359-010-0614-5).

Neuweiler G. 1989 Foraging ecology and audition in echolocating bats. *Trends Ecol Evol* **4**(6), 160-166. (doi:10.1016/0169-5347(89)90120-1).

Neuweiler G., Singh S., Sripathi K. 1984 Audiograms of a South Indian bat community. *J Comp Physiol, A* **154**(1), 133-142. (doi:10.1007/BF00605398).

Obrist M.K., Wenstrup J.J. 1998 Hearing and hunting in red bats (*Lasiurus borealis*, Vespertilionidae): audiogram and ear properties. *J Exp Biol* **201**(1), 143-154.

Petrites A.E., Eng O.S., Mowlds D.S., Simmons J.A., DeLong C.M. 2009 Interpulse interval modulation by echolocating big brown bats (*Eptesicus fuscus*) in different densities of obstacle clutter. *J Comp Physiol, A* **195**(6), 603-617. (doi:10.1007/s00359-009-0435-6).

Pollak G., Henson O., Johnson R. 1979 Multiple specializations in the peripheral auditory system of the CF-FM bat, *Pteronotus parnellii*. *J Comp Physiol, A* **131**(3), 255-266. (doi:10.1007/BF00610433).

Ratcliffe J.M., Dawson J.W. 2003 Behavioural flexibility: the little brown bat, *Myotis lucifugus*, and the northern long-eared bat, *M. septentrionalis,* both glean and hawk prey. *Anim Behav* **66**(5), 847-856. (doi:10.1006/anbe.2003.2297).

Rodríguez-San Pedro A., Allendes J.L. 2017 Echolocation calls of free-flying common vampire bats *Desmodus rotundus* (Chiroptera: Phyllostomidae) in Chile. *Bioacoustics* **26**(2), 153-160. (doi:10.1080/09524622.2016.1231079).

Rübsamen R. 1987 Ontogenesis of the echolocation system in the rufous horseshoe bat, *Rhinolophus rouxi* (audition and vocalization in early postnatal development). *J Comp Physiol, A* **161**(6), 899-913. (doi:10.1007/BF00610231).

Rübsamen R., Neuweiler G., Sripathi K. 1988 Comparative collicular tonotopy in two bat species adapted to movement detection, *Hipposideros speoris* and *Megaderma lyra*. *J Comp Physiol, A* **163**(2), 271-285. (doi:10.1007/BF00612436).

Schmidt U., Schlegel P., Schweizer H., Neuweiler G. 1991 Audition in vampire bats, *Desmodus rotundus*. *J Comp Physiol, A* **168**(1), 45-51. (doi:10.1007/BF00217102).

Schmidt U.v., Manske U. 1973 Die Jugendentwicklung der Vampirfledermäuse (*Desmodus rotundus*). *Z Säugetierkd* **38**, 14-33.

Schmidt-French B., Gillam E., Fenton M.B. 2006 Vocalizations emitted during mother-young interactions by captive eastern red bats *Lasiurus borealis* (Chiroptera: Vespertilionidae). *Acta Chiropt* **8**(2), 477-484. (doi:10.3161/1733-5329(2006)8[477:VEDMIB]2.0.CO;2).

Schnitzler H.-U., Kalko E.K., Kaipf I., Grinnell A.D. 1994 Fishing and echolocation behavior of the greater bulldog bat, *Noctilio leporinus*, in the field. *Behav Ecol Sociobiol* **35**(5), 327-345. (doi:10.1007/BF00184422).

Schuller G. 1980 Hearing characteristics and Doppler shift compensation in South Indian CF-FM bats. *J Comp Physiol, A* (4), 349-356. (doi:10.1007/BF00610465).

Siemers B.M., Kalko E.K., Schnitzler H.-U. 2001 Echolocation behavior and signal plasticity in the Neotropical bat *Myotis nigricans* (Schinz, 1821)(Vespertilionidae): a convergent case with European species of *Pipistrellus*? *Behav Ecol Sociobiol* **50**(4), 317-328. (doi:10.1007/s002650100379).

Simmons J.A., Kick S.A., Lawrence B.D. 1984 Echolocation and hearing in the mouse-tailed bat, *Rhinopoma hardwickei:* acoustic evolution of echolocation in bats. *J Comp Physiol, A* **154**(3), 347-356. (doi:10.1007/BF00605234).

Simon R., Holderied M.W., Von Helversen O. 2006 Size discrimination of hollow hemispheres by echolocation in a nectar feeding bat. *J Exp Biol* **209**(18), 3599-3609. (doi:10.1242/jeb.02398).

Sterbing S.J., Schmidt U., Rübsamen R. 1994 The postnatal development of frequency-place code and tuning characteristics in the auditory midbrain of the phyllostomid bat, *Carollia perspicillata*. *Hearing Res* **76**(1-2), 133-146. (doi:10.1016/0378-5955(94)90095-7).

Thies W., Kalko E.K., Schnitzler H.-U. 1998 The roles of echolocation and olfaction in two Neotropical fruit-eating bats, *Carollia perspicillata* and *C. castanea*, feeding on *Piper*. *Behav Ecol Sociobiol* **42**(6), 397-409. (doi:10.1007/s002650050454).

Thomson C., Fenton M., Barclay R. 1985 The role of infant isolation calls in mother–infant reunions in the little brown bat, *Myotis lucifugus* (Chiroptera: Vespertilionidae). *Can J Zool* **63**(8), 1982-1988. (doi:10.1139/z85-290).

Vater M., Kossl M., Foeller E., Coro F., Mora E., Russell I. 2003 Development of echolocation calls in the mustached bat, *Pteronotus parnellii*. *J Neurophysiol* **90**(4), 2274-2290. (doi:10.1152/jn.00101.2003).

Waters D.A., Jones G. 1995 Echolocation call structure and intensity in five species of insectivorous bats. *J Exp Biol* **198**(2), 475-489.

Wenstrup J.J. 1984 Auditory sensitivity in the fish-catching bat, *Noctilio leporinus*. *J Comp Physiol, A* **155**(1), 91-101. (doi:10.1007/BF00610934).

Wetekam J., Reissig C., Hechavarria J.C., Kössl M. 2020 Auditory brainstem responses in the bat *Carollia perspicillata*: threshold calculation and relation to audiograms based on otoacoustic emission measurement. *J Comp Physiol, A* **206**(1), 95-101. (doi:10.1007/s00359-019-01394-6).

Wittekindt A., Drexl M., Kössl M. 2005 Cochlear sensitivity in the lesser spear-nosed bat, *Phyllostomus discolor*. *J Comp Physiol, A* **191**(1), 31-36. (doi:10.1007/s00359-004-0564-x).
